# Methylation studies in *Peromyscus*: aging, altitude adaptation, and monogamy

**DOI:** 10.1007/s11357-021-00472-5

**Published:** 2021-10-26

**Authors:** Steve Horvath, Amin Haghani, Joseph A. Zoller, Asieh Naderi, Elham Soltanmohammadi, Elena Farmaki, Vimala Kaza, Ioulia Chatzistamou, Hippokratis Kiaris

**Affiliations:** 1 Department of Human Genetics, David Geffen School of Medicine, University of California, Los Angeles, CA USA; 2 Department of Biostatistics, Fielding School of Public Health, University of California, Los Angeles, CA USA; 3 Department of Drug Discovery and Biomedical Sciences, College of Pharmacy, University of South Carolina, Columbia, SC USA; 4 Peromyscus Genetic Stock Center, University of South Carolina, Columbia, SC USA; 5 Department of Pathology, Microbiology and Immunology, School of Medicine, University of South Carolina, Columbia, SC USA

**Keywords:** Deer mouse, Aging, Epigenetic clock, DNA methylation

## Abstract

DNA methylation-based biomarkers of aging have been developed for humans and many other mammals and could be used to assess how stress factors impact aging. Deer mice (*Peromyscus*) are long-living rodents that have emerged as an informative model to study aging, adaptation to extreme environments, and monogamous behavior. In the present study, we have undertaken an exhaustive, genome-wide analysis of DNA methylation in *Peromyscus*, spanning different species, stocks, sexes, tissues, and age cohorts. We describe DNA methylation-based estimators of age for different species of deer mice based on novel DNA methylation data generated on highly conserved mammalian CpGs measured with a custom array. The multi-tissue epigenetic clock for deer mice was trained on 3 tissues (tail, liver, and brain). Two human-*Peromyscus* clocks accurately measure age and relative age, respectively. We present CpGs and enriched pathways that relate to different conditions such as chronological age, high altitude, and monogamous behavior. Overall, this study provides a first step towards studying the epigenetic correlates of monogamous behavior and adaptation to high altitude in *Peromyscus*. The human-*Peromyscus* epigenetic clocks are expected to provide a significant boost to the attractiveness of *Peromyscus* as a biological model.

**Supplementary Information:**

The online version contains supplementary material available at 10.1007/s11357-021-00472-5.

## Introduction

Cytosine methylation is an epigenetic mechanism which plays a critical role in mammalian development [1]. Methyl groups may help to generate a local chromatin configuration that renders genes inaccessible, and thus transcriptionally inactive [2]. In general, cytosine methylation at specific sites is influenced by both heritable as well as environmental factors [3]. It has long been recognized that age has a strong effect on DNA methylation (DNAm) levels [4–6]. DNA methylation data can be used to develop multivariate regression models that accurately estimate chronological age for any tissue across the entire lifespan of mammals [7–13]. These DNAm-based age estimators, also known as epigenetic clocks, use penalized regression models to predict chronological age based on DNA methylation levels (reviewed in [13, 14]). Methylation stands out from other genomic data in that it allows one to build pan-tissue clocks that apply to all tissue types across the entire lifespan (from prenatal samples to centenarians) [11, 12, 15].

Species from the genus *Peromyscus* (deer mouse) are appealing models for addressing various biological questions in relation to aging because they live up to 8 years in captivity [16]*,* a lifespan that exceeds by about threefold, that of animals of the genus Mus, the commonly used model for biomedical research. They are also used to study metabolism, infectious diseases, adaptation at extreme environments, such as high altitude and the desert, monogamous behavior, as well behavioral responses in response to anxiety stress [16–19]. In an effort to better understand how methylation profiles change in relation to different environments and genetic backgrounds, we have undertaken to study methylation patterns in the genus *Peromyscus* spanning different species and stocks/populations. *Peromyscus* is comprised of species that were evolved in diverse range habitats, from deserts to high-altitude mountains, in environments with low-to-high extrinsic risk of predation. Thus, each one of these species gained several unique features to adapt, reproduce, and survive in these environments. Here, we present a comparative epigenetic analysis of these interesting characteristics. We characterize CpGs that relate to mating behavior (e.g., monogamy) and high/low altitude.

Further, we present six highly accurate epigenetic clocks for Peromyscus. Two of these clocks apply to humans as well.

## Results

### Data sets

Different species of *Peromyscus* are maintained as closed colonies of outbred, genetically diverse stocks at the *Peromyscus* Genetic Stock Center. The present analysis involved 36,000 CpGs that are highly conserved across mammals in DNA and was applied to specimens from two *Peromyscus* subgenera, 5 species and one interspecific hybrid, 3 tissues (tails, brain, and liver), and individuals from ages ranging from 2 months to about 3.6 years. The choice of tissues was informed by several criteria. First, the Mammalian Methylation Consortium aims to collect liver samples and brain samples from as many species as possible for the sake of comparative studies. For these tissues, abundant methylation data are available from various species, allowing the cross-species evaluation of data generated. Second, we also aimed to profile a tissue that could be collected without sacrificing the animal (tail).

The specimens analyzed involved both males and females as well as individuals from two different closed colonies of *P. maniculatus*. This experimental setup facilitated a simultaneous, unbiased analysis of DNA methylation signatures in specimens differing across different levels of biological organization.

**Fig. 1:**
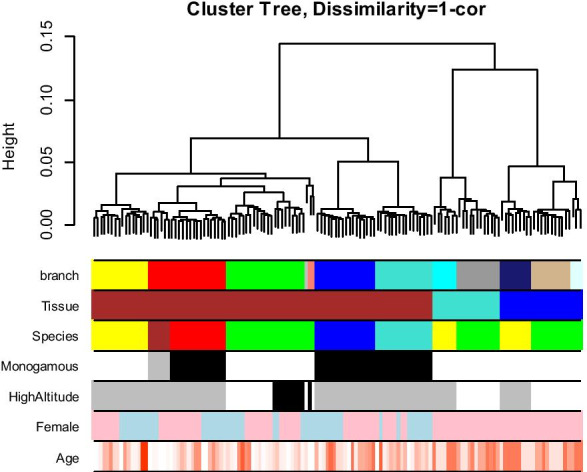
Unsupervised hierarchical clustering of tissue samples from deer mice. Average linkage hierarchical clustering based on the interarray correlation coefficient (Pearson correlation). The first color band is based on cutting the branches at a height cutoff value of 0.03. Note that the branch colors correspond to tissue (second color band) and species (third color band). Tissue encodes tail (brown), whole brain (turquoise), and liver (blue). Species encodes *Peromyscus californicus* (turquoise), *eremicus* (blue), *leucopus* (yellow), *maniculatus* (green), *polionatus* (red), and a hybrid between *polionatus* and *maniculatus* (brown). The fourth color band encodes monogamous (black) versus polygamous (white) behavior. The fifth color band encodes high altitude population (black) versus low altitude (white). Female (pink). Age color codes old (red) versus young (white)

We used a custom methylation array (HorvathMammalMethylChip40) to generate DNA methylation data from six species of *Peromyscus*: *Peromyscus californicus* (*n* = 16), *Peromyscus eremicus* (*n* = 17), *Peromyscus* hybrid between *P. polionotus* and *P. maniculatus* (*n* = 6), *Peromyscus leucopus* (*n* = 36), *Peromyscus maniculatus* (*n* = 53), and *Peromyscus polionotus* (*n* = 16). Hierarchical clustering indicated the *n* = 6 samples were technical outliers (Fig. 1). These samples were subsequently removed from the analysis.

These DNA samples came from three tissues/organs: whole brain, tail, and liver as detailed in Table 1. The ages ranged from 0.083 to 3.6 years. Additionally, we used DNA methylation profiles from 1205 human samples, from several tissues, and with a large age range, to construct two dual-species human-*Peromyscus* epigenetic clocks. These human data were generated on the same custom methylation array, which was designed to facilitate cross-species comparisons across mammals.

**Table 1:** Description of the data. *n* = total number of tissues. Number of females. Age (in units of years): mean, minimum, and maximum age

Species/tissue	*n*	No. of females	Mean age	Min. age	Max. age
*Peromyscus californicus*/tail	16	7	0.955	0.083	2.75
*Peromyscus eremicus*/tail	17	9	1.36	0.16	2.66
Hybrid polionotus + maniculatus/tail	6	3	0.163	0.083	0.25
*Peromyscus leucopus*/brain	7	7	1.58	0.583	2.33
*Peromyscus leucopus*/liver	8	8	1.46	0.583	2.33
*Peromyscus leucopus*/tail	16	8	1.13	0.083	3.58
*Peromyscus maniculatus*/brain	12	12	1.58	0.583	2.42
*Peromyscus maniculatus*/liver	13	13	1.46	0.583	2.42
*Peromyscus maniculatus*/tail	25	14	0.724	0.083	2.83
*Peromyscus polionotus*/tail	16	9	0.909	0.083	1.91

### Unsupervised hierarchical clustering of deer mouse methylation data

Unsupervised hierarchical clustering of methylation data was initially performed that identified the tissue identity as the most prominent discriminator of global methylation signatures (Fig. 1). Thus, the profile of DNA methylation is primarily guided by a function which, in turn, underscores the impact of this epigenetic modification in regulating gene transcription and therefore guiding cellular differentiation. Within the same tissue, clustering occurred in a manner that overlapped astonishingly with the evolutionary history. Initially, two branches emerged, with the first including *P. californicus* and *P. eremicus* and the second comprised of *P. leucopus*, *P. maniculatus*, and *P. polionotus*, that signify two distinct groups in the evolution of *Peromyscus* [20]. Among them, the highly related *P. maniculatus* and *P. polionotus* clustered together, while *P. leucopus* has diverged earlier. Indeed*, P. maniculatus* and *P. polionotus* can form interspecific F1 hybrids that, in relation to their methylation patterns, are more closely related to the paternal strain, *P. polionotus* (Fig. 1**)** pointing to the impact of the parent of origin effects in guiding methylation signatures [21]. Among the same species, genetic relevance appeared to be of significance. Individuals from two closed *P. maniculatus* colonies were evaluated, *bairdii* and *sonoriensis* (BW and SM2 stocks, respectively) [16]. Individuals from each of these colonies clustered accurately together suggesting that genetic relatedness is capable of triggering similar patterns of DNA methylation that surpass those inflicted by the sex and the age of the individuals from which the DNA samples have been isolated. Within the same species and stocks, clustering occurred according to sex. The fact that the analysis involved tails, whole brain, and liver samples that are not major target tissues for sex hormones implies that sex-specific patterns of methylation are inflicted early during development that persist at adulthood. Alternatively, the expression of receptors for gonadal steroids by these tissues may have caused modulation of the methylation profile and the observed clustering of the specimens according to sex; nevertheless, this appears to be of lesser impact as compared to the other variables examined.

### Epigenetic clocks

**Fig. 2:**
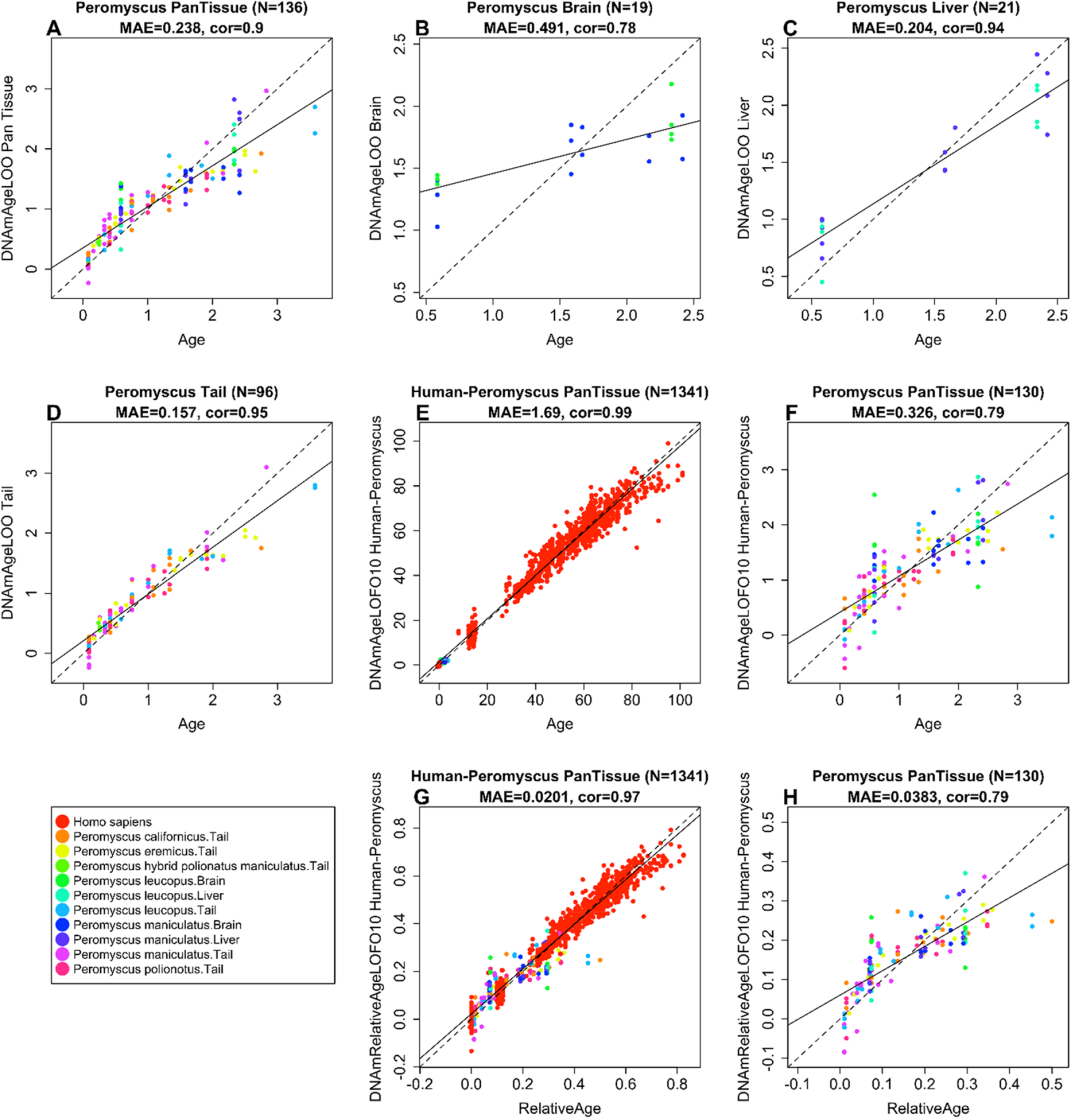
Cross-validation study of epigenetic clocks for *Peromyscus*. The *y*-axis reports the DNA methylation estimate of **A**–**F** chronological age (age in units of years) or **G** and **H** relative age. **a** Epigenetic clock for multiple *Peromyscus* species. Leave-one-sample-out (LOO) estimate of DNA methylation age (*y*-axis, in units of years) versus chronological age. **A** Multi-tissue clock for all considered *Peromyscus* species, **B** brain clock, **C** liver clock, and **D** tail clock. **E** Dual species human-*Peromyscus* clock for chronological age, **F** excerpt of panel **E** restricted to *Peromyscus* samples. **G** Human-*Peromyscus* clock for relative age (defined as the ratio of chronological age to maximum lifespan). **H** Excerpt of panel **G** restricted to *Peromyscus* samples. Dots (DNA samples) are colored by species or tissue sample as outlined in the legend. Each panel reports the sample size, correlation coefficient, and median absolute error (MAE)

Our different clocks can be distinguished along two dimensions (species and measure of age). The multi-tissue clock (also referred to as pan-tissue clock) for *Peromyscus* was developed by regressing chronological age on CpGs in all available tissue samples from all species. Since the regression model was fit to different *Peromyscus* species, the resulting age estimators are multi-species clocks that are expected to apply to all *Peromyscus* species. To arrive at unbiased estimates of our DNA methylation-based age estimators, we performed a cross-validation study in the training data. The cross-validation study reports unbiased estimates of the age correlation *R* (defined as Pearson correlation between the age estimate (DNAm age) and chronological age as well as the median absolute error. The multi-tissue clock leads to a high correlation between estimated age and actual age (cross-validation estimate of the Pearson correlation *R* = 0.9, median absolute error = 0.24 years = 3 months, Fig. 2a). Similarly, we developed tissue-specific clocks by focusing on one specific tissue type. Since multiple Peromyscus species were used, the resulting clocks should again be interpreted as multispecies clocks. Cross-validation studies indicate high accuracy for the brain clock (cross-validation estimate *R* = 0.78, MAE = 0.49 years, Fig. 2b), liver clock (*R* = 0.94, MAE = 0.20, Fig. 2c), and tail clock (*R* = 0.95, MAE = 0.16, Fig. 2d).

Evolutionarily speaking, primates are quite distant from *Peromyscus*. However, we managed to build two human-*Peromyscus* multi-tissue clocks. The first human-*Peromyscus* clock estimates chronological age using a single multivariate regression model. We observe high correlation coefficients between age and its estimates across both species (cross-validation estimate *R* = 0.99, Fig. 2e) and when the analysis is restricted to *Peromyscus* samples (*R* = 0.79, Fig. 2f). The second human-*Peromyscus* clock estimates relative age, defined as the ratio of chronological age to maximum lifespan. This clock achieves a similar performance (*R* = 0.97 and *R* = 0.79, Fig. 2g,h). By definition, the relative age takes values between 0 and 1 and arguably provides a biologically more meaningful comparison between species with different lifespans (deer mouse and human), which is not afforded by mere measurement of the absolute age.

Peromyscus are long-living rodents that exhibit variable lifespan depending on the species [22]. According to the database “anAge” [23], the maximum lifespans are as follows: 7.4 years for *Peromyscus eremicus* [24], 7.9 years for *Peromyscus leucopus* [24], 8.3 years for *Peromyscus maniculatus* [25], and 5.5 years for *Peromyscus polionotu*s [24]. While the maximum lifespan estimates may be debatable, our clock is quite robust with respect to different choices of this mathematical parameter. Similarly, accurate clocks could be constructed with different parameter choices.

### EWAS of age

In total, 29,125 probes from the mammalian chip (HorvathMammalMethylChip40) were aligned to specific loci approximate to 5048 genes in the deer mouse genome (Peromyscus_maniculatus_bairdii.HU_Pman_2.1.100). These probes have high conservation in mammals, thus comparable among different species. In our epigenome-wide association study (EWAS) of age, we correlated individual CpGs to chronological age. Stratified results for each deer mouse species and tissue type are presented in Supplementary Fig. 1. Significant age-related CpGs were located in both genic and intergenic regions relative to the transcriptional start site. CpGs located in promoters and CpG islands gained methylation with age.

For comparative purposes, we contrasted these results to those from an EWAS of chronological age in liver and tail data from C57Bl/6 mice (*Mus musculus*). We caution the reader that C57Bl/6 mice are inbred, unlike our deer mice. In general, EWAS of age in tails and liver from *Peromyscus* species were only weakly related to the corresponding analyses of the same tissues from C57Bl/6 mice. Age effects in liver methylation data from C57Bl/6 mice were only weakly correlated with those from *P. maniculatus* (*R* = 0.16, Supplementary Fig. 3) and *P leucopus (R* = 0.32*).* Age effects in tail methylation data from C57Bl/6 mice were only weakly correlated with those from *P. maniculatus* (*R* = 0.12, Supplementary Fig. 4), *P leucopus (R* = *0.47), P. polionotus* (*R* = 0.32).

**Fig. 3:**
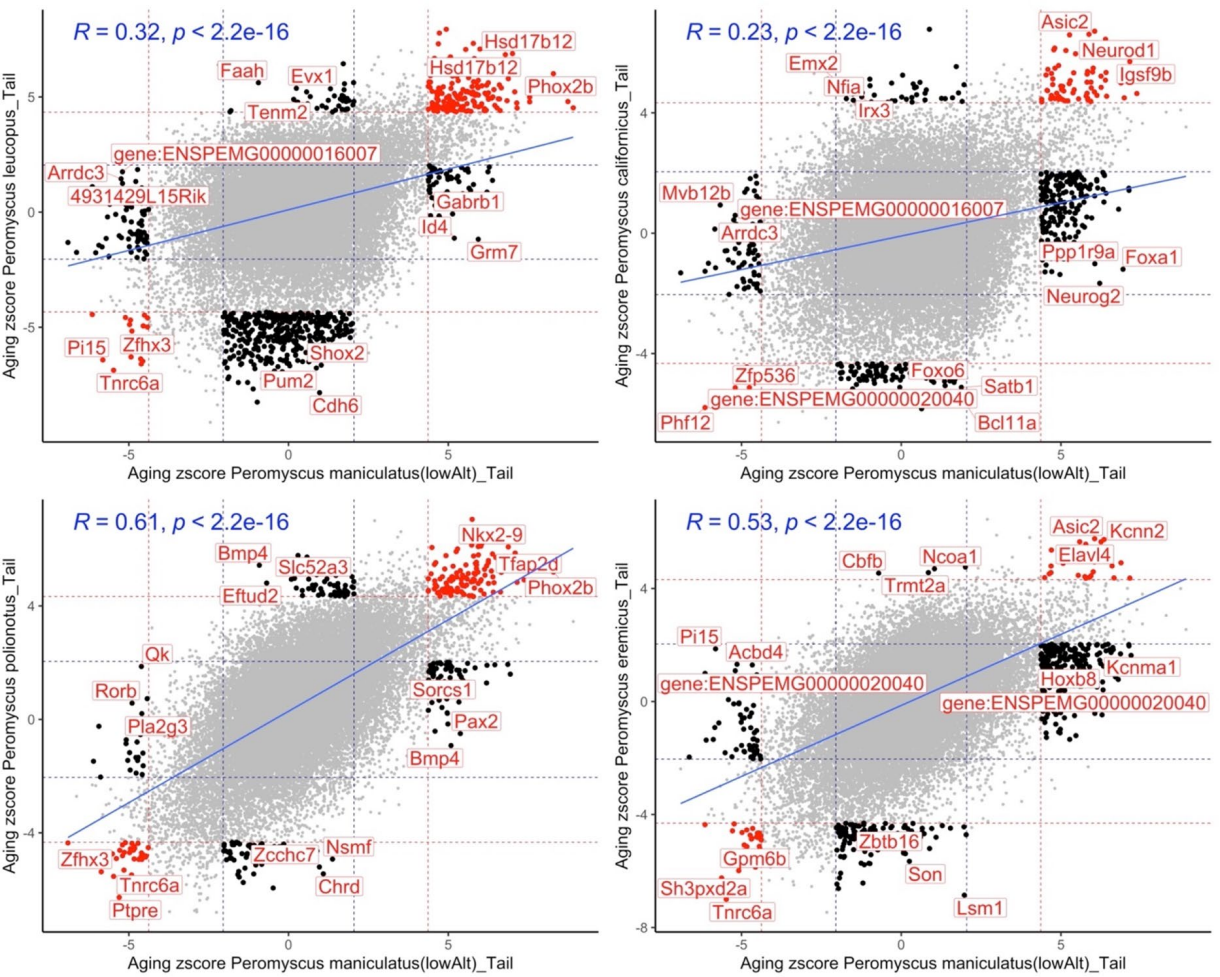
Comparative DNAm aging between *P. maniculatus* and four other *Peromyscus* species. Scatter plots represent the EWAS of age in *P. maniculatus* (*x*-axis) vs other four species. The aging *Z* statistics are the Fisher *Z*-transformation of DNAm-age Pearson correlation for each CpG in the tail samples of each species. A positive (or decrease) of *Z* statistics means an increase (or decrease) of DNAm by age in the analyzed species. Red dotted lines are the *Z* statistics corresponding to *p* < 10^−4^; blue dotted lines are the *Z* statistics corresponding to *p* > 0.05; red dots indicate the shared CpGs (i.e., The CpGs that significantly change in the same direction in both species) between x and y axes; black dots: the changes that are significant in one species but not the other. The top CpGs in each sector are labeled by the adjacent gene based on the *P. maniculatus* genome. *Peromyscus maniculatus* (lowAlt) is the *P. maniculatus* originated from the founders with a low altitude habitat. Sample size: *Peromyscus maniculatus* (lowAlt), 40; *Peromyscus leucopus*, 31; *Peromyscus californicus*, 16; *Peromyscus polionotus*, 16; *Peromyscus eremicus*, 17

In a separate analysis, we compared epigenetic aging effects in *P. maniculatus* with those in four different *Peromyscus* species (Fig. 3). Pairwise correlations differed within these species which probably reflects evolutionary differences of these highly related species. Despite these differences, there were numerous CpGs with consistent aging patterns in the *Peromyscus* genome. Some of the shared changes in these animals included a gain of methylation in *Bdnf* (promoter), *Igsf9b* (exon), *Nkx2-9* (downstream), *Lhfpl4* (exon), *Elavl4* (intron), and *Trhde* (promoter).

### Epigenetic profile of North American deer mouse change along with the altitude

**Fig. 4:**
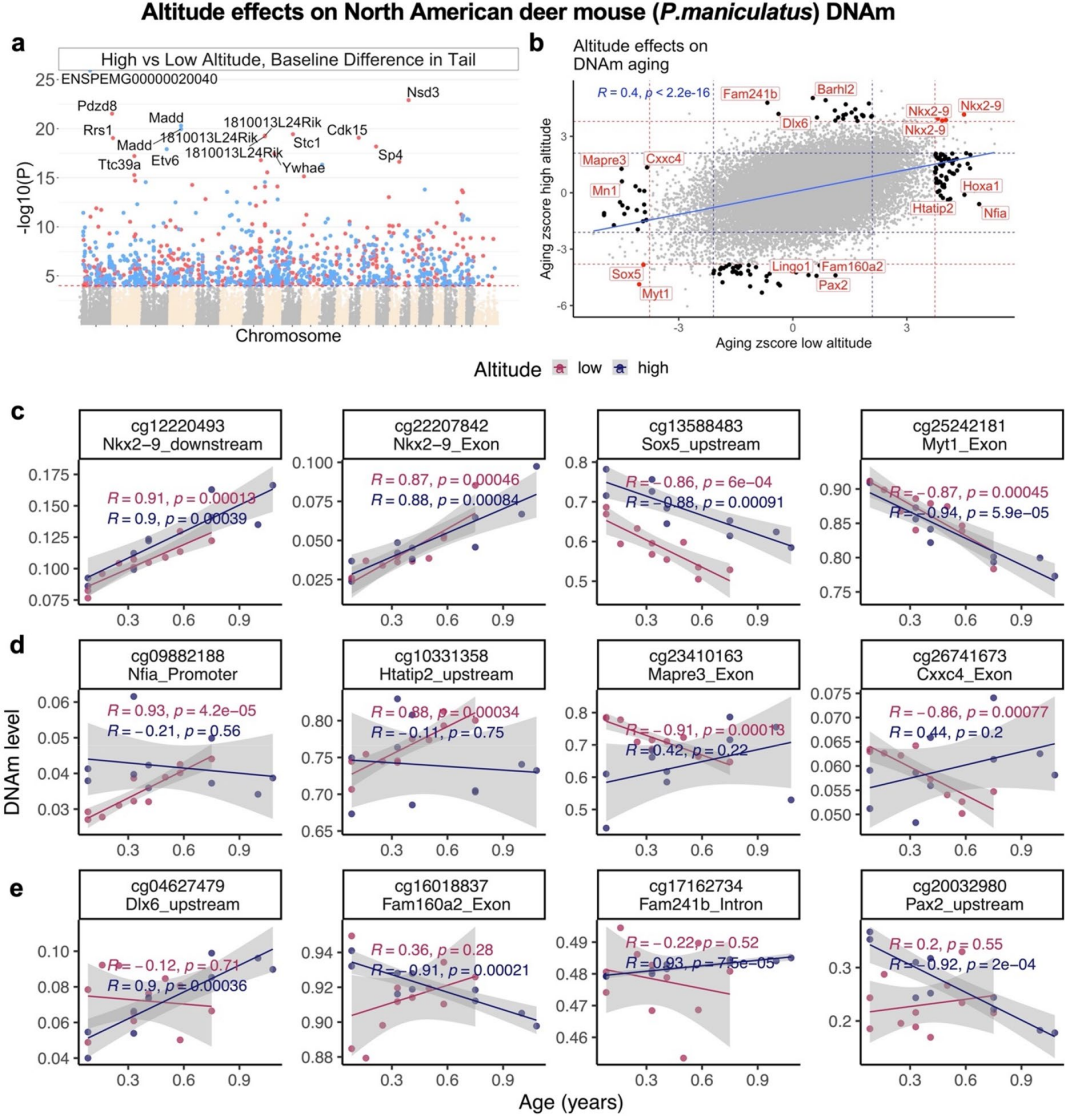
Altitude difference altered epigenetic profiles of *P. maniculatus*. This study compares two colonies of *P. maniculatus*, which originated from the founders with different habitats in different altitudes. **a** The Manhattan plot of EWAS of altitude in the tail of *P. maniculatus* species after adjusting for chronological age. The genome coordinates are estimated based on the alignment of mammalian array probes to the Peromyscus_maniculatus_bairdii.HU_Pman_2.1.100 genome assembly. The direction of associations with *p* < 10^−4^ (red dotted line) is colored in red (increased methylation) and blue (decreased methylation). The 15 most significant CpGs are labeled by adjacent genes. **b** Sector plot of DNA methylation aging effects in *P. maniculatus* species from different altitudes. The *Z* statistics for aging effects result from applying the Fisher *Z*-transformation to the Pearson correlation between age and the respective CpG in the tail samples of each colony. The red and blue dotted lines correspond to a *p*-value threshold of *p* < 0.01 and *p* < 0.05, respectively. Shared CpGs (i.e., the CpGs that significantly change in the same direction) are color-coded in red. Black dots correspond to CpGs that are significant in one colony but not the other. The scatter plots represent the CpGs that change with age in both (**c**), or uniquely in low (**d**) or high (**e**) altitude colonies. The adjacent gene region of each CpG is reported in the title of each plot. The shade around the lines is the 95% confidence interval based on the linear regression model. *R*: Pearson correlation coefficient

The present analysis involved individuals from two colonies of *P. maniculatus sonoriensis* (SM2 stock) and *P. maniculatus bairdii* (BW stock). The original founders of these colonies have evolved in high- or low-altitude environments, respectively, and since then are maintained as closed colonies. We identified a total of 1273 CpGs that differed in their mean methylation levels at a nominal significance threshold of *p* < 10^−4^ (Fig. 4a).

CpGs in *ENSPEMG00000020040* (downstream, *p* = 1 × 10^−26^) and *Madd* (exon, *p* = 3 × 10^−17^) show decreased methylation in the high-altitude stock *P. maniculatus sonoriensis* while CpGs in *Nsd3* (intron), *Pdzd8* (exon, *p* = 2.9 × 10^−22^), and *Stc1* (promoter, *p* = 5 × 10^−20^) show increased methylation (Fig. 4a). Functional enrichment studies of the top 500 CpGs per direction (increased/decreased) showed that these loci tend to be located near genes that play a role in the development of rhombomere 3 (*p* = 3.9 × 10^−10^), motor neurons (*p* = 2 × 10^−7^), middle ear (*p* = 5 × 10^−10^), immune system functioning (p = 2.8 × 10^−11^), antigen processing, and presentation (p = 9.6 × 10^−6^).

Next, we examined the differences in aging patterns, i.e., differences in the age correlations between the two colonies of *P. maniculatus*. Aging effects showed a moderately high correlation between the two colonies (*R* = 0.4, Fig. 4b,c), but several CpGs exhibited significant correlations only in one of the colonies. DNAm aging effect comparison by altitude was done at a nominal *p* < 10^−3^ in order to implicate sufficient numbers of CpGs for our subsequent enrichment studies.

For example, CpGs in the promoter of *Nfia*, upstream of *Htatip2*, in an exon of *Mapre3*, and an exon of *Cxx4* correlated significantly with age only in the low altitude colony of *P. maniculatus*. Conversely, CpG upstream of *Dlx6*, an exon of *Fam160a2*, an intron of *Fam241b*, and upstream of *Pax2* correlated significantly with age only in the high-altitude colony (Fig. 4d,e).

Interestingly, these altitude-specific age-related changes are adjacent to genes that play a role in brain development (*p* = 2.7 × 10^−4^), immune system functioning (*p* = 4.6 × 10^−5^), and T-cell development (*p* = 4.6 × 10^−5^). Overall, these results suggest that altitude affects both brain development and immune system functioning. Limitations of the analysis are discussed below.

### Brain aging patterns in North American deer mouse species

**Fig. 5:**
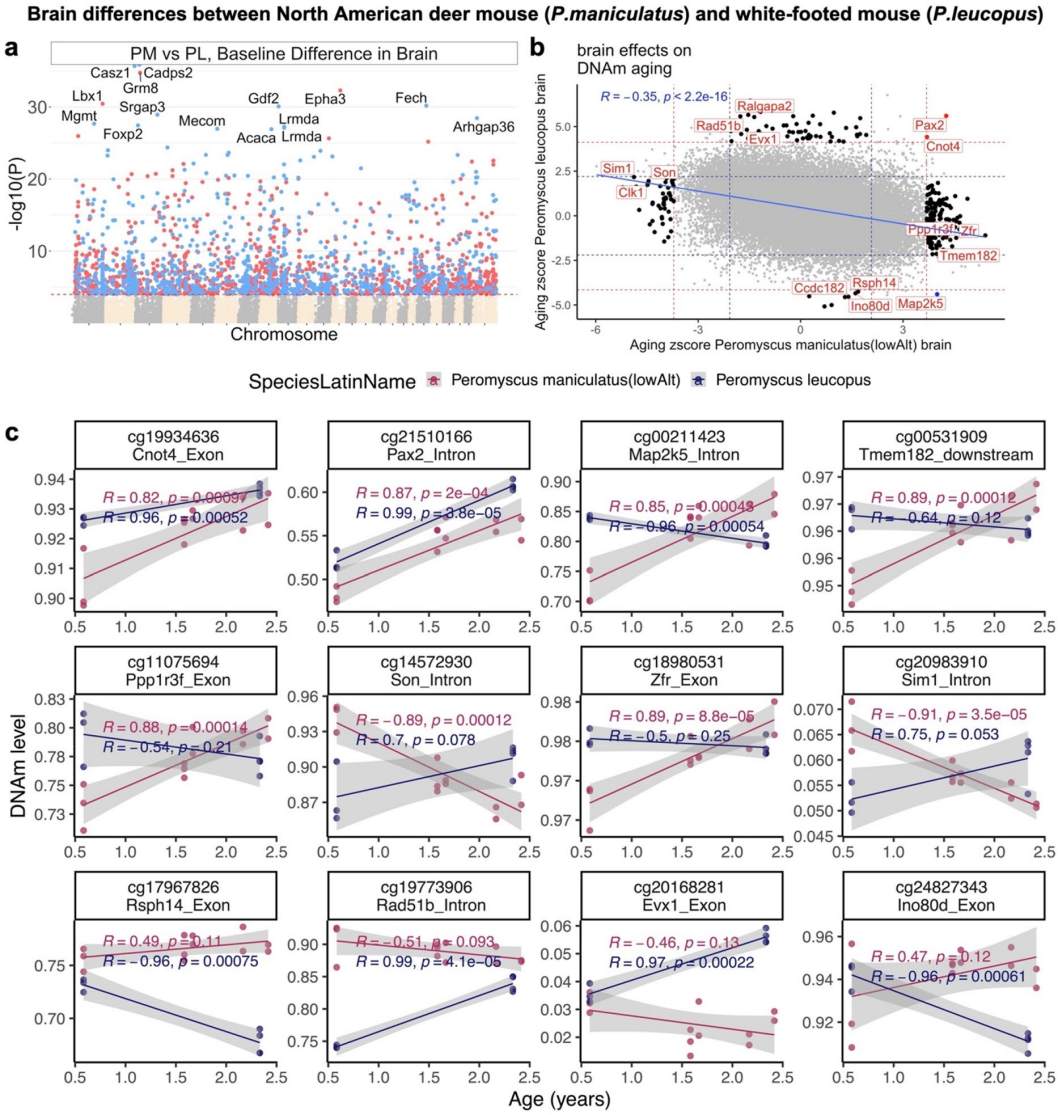
Brain methylation differences between *P. maniculatus* (PM) and *P. leucopus* (PL). **a** The Manhattan plot of mean methylation differences between *P. maniculatus* and *P. leucopus* after adjusting for chronological age. Genome coordinates for the Peromyscus_maniculatus_bairdii.HU_Pman_2.1.100 genome assembly. The direction of associations with *p* < 10^−4^ (red dotted line) is colored in red (hypermethylated) or blue (hypomethylated). The top 15 CpGs are labeled by adjacent genes. **b** Sector plot of DNAm aging effects in *P. maniculatus* and *P. leucopus*. The aging *Z* statistics are the Fisher *Z*-transformation of DNAm-age Pearson correlation for each CpG in the tail samples of each species. The red and blue dotted lines correspond to a *p*-value threshold of *p* < 0.01 and *p* < 0.05, respectively. Red color codes shared CpGs (i.e., the CpGs that significantly change in the same direction) while black color code changes that are significant in one species but not the other. **c** The scatter plots represent the CpGs that change with age in both or uniquely in one of these species. The adjacent gene region of each CpG is reported in the title of each plot. The shade around the lines is the 95% linear regression confidence interval. *R*: Pearson correlation coefficient

Comparison of brain specimens between older *P. leucopus* and *P. maniculatus* indicated that in the latter, coordination of the unfolded protein response is compromised, and evidence of neurodegenerative pathology was obtained [26]. Therefore, comparative analysis of *Peromyscus* species may be relevant to the study of age-related alterations in the brain. Here, we compared the DNAm profile of the *P. maniculatus* brain to *P. leucopus* and found strong differences. A total number of 2396 CpGs were differentially methylated between these two species at *p* < 10^−4^ (Fig. 5a). In *P. maniculatus*, CpGs were hypomethylated in *Cadsp2* (exon, *p* = 1.3 × 10^−36^), *Casz1* (exon, *p* = 3 × 10^−32^) but hypermethylated in *Grm8* (exon, *p* = 1.8 × 10^−35^), *Epha3* (promoter, *p* = 5 × 10^−33^), and *Lbx1* (promoter, *p* = 3.5 × 10^−31^). GREAT enrichment analysis of the top 500 most significant CpGs in each direction revealed that these CpGs were adjacent to genes that play a role in the circadian rhythm (GREAT hypergeometric *p* = 9 × 10^−12^, Supplementary Fig. 6), histone H2A ubiquitination (*p* = 6 × 10^−8^), and glucagon secretion (*p* = 2.7 × 10^−7^). The latter finding surrounding glucagon secretion is interesting since several brain regions are known to be sensitive to glucagon by induction of cAMP signaling cascades [27], and insulin resistance may be related to Alzheimer’s disease [28].

Strikingly, age effects in brain methylation data from *P. maniculatus* were inversely correlated (*R* =  − 0.35) with those in *P. leucopus* brains (Fig. 5b). We could only identify two CpGs (adjacent to *Cnot4* exon, *Pax2* intron) that changed with age in the same direction in these species (Fig. 5c). In contrast, several brain aging loci were identified that only changed in one of these species, or even diverged during aging. *Map2k5* intron was an extreme example that was hypermethylated in *P. maniculatus* but hypomethylated in *P. leucopus* (Fig. 5b,c). CpGs that were only significantly correlated with age in *P. leucopus* were enriched in angiogenesis-related processes (*p* = 2.3 × 10^−4^) while those unique to *P. maniculatus* were enriched in gamma delta T cells (p = 9 × 10^−4^). However, the latter findings could be false positives since the enrichment *p*-values are not significant after adjusting for multiple comparisons (nominal *p*-value > 10^−4^).

### DNAm relate to pair-bonding behavior in deer mouse species

**Fig. 6:**
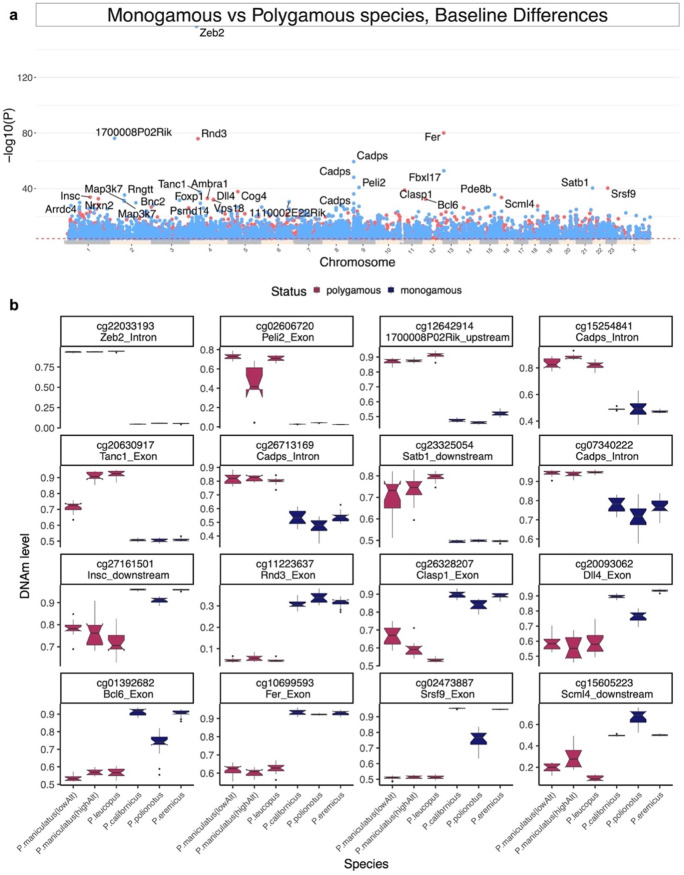
CpGs that differ between monogamous and polygamous *Peromyscus* species. **a** The Manhattan plot of the mean DNAm difference between monogamous and polygamous *Peromyscus* species after adjusting the analysis for chronological age in a tail sample of *Peromyscus* species. Monogamous species: *P. californicus* (*N* = 16), *P. polionotus* (*N* = 16), *P. eremicus* (*N* = 16). Polygamous species: *P. maniculatus* (both altitude colonies, *N* = 25), *P. leucopus* (*N* = 16). Genome coordinates from Peromyscus_maniculatus_bairdii.HU_Pman_2.1.100 genome assembly. The direction of associations with *p* < 10^−3^ (red dotted line) is colored in red (hypermethylated) and blue (hypomethylated). The top 15 CpGs are labeled by their neighboring genes. **b** The box plot of the top CpGs that differed by dyadic relationship in *Peromyscus* species. The adjacent gene region of each CpG is reported in the title of each boxplot. The notches in the boxplots indicate the 95% confidence interval of the median DNAm values for each group

Relatively, few mammalian species are monogamous [29]. Pair bonds based on mating, that are associated with the development of monogamous behavior are estimated to occur in less than 10% of mammals, including humans [30, 31]. In *Peromyscus*, monogamous behavior is fairly common and has developed independently at least twice in evolution [30, 32]. Our study involved three monogamous (*P. californicus*, *P. polionotus*, and *P. eremicus*) and two polygamous (*P. maniculatus* and *P. leucopus*) species. Monogamous species differed greatly from polygamous species: 9411 CpGs were differentially methylated at a nominal significance threshold of *p* < 10^−4^ (Fig. 6a). The most significant EWAS hits for monogamy included decreased methylation in *Zeb2* intron (*p* = 1.8 × 10^−153^), *1700008P02Rik* upstream (*p* = 6.2 × 10^−77^), *Cadps* intron (*p* = 4.1 × 10^−60^), and increased methylation in *Fer* exon (*p* = 9.2 × 10^−76^), *Rnd3* exon (*p* = 1.24 × 10^−76^), and *Srsf9* exon (*p* = 4.9 × 10^−41^) (Fig. 6b). For enrichment analysis, we limited the analysis to the top 500 CpGs per direction (increased/decreased). While, a monogamous-related decrease of methylation was enriched with synaptic dopamine release (*p* = 8 × 10^−4^) in the mouse phenotype database, the increase of methylation was associated with immune-related biological processes such as antigen processing and presentation by MHCII cells (*p* = 1 × 10^−4^) (Supplementary Fig. 7). Dopamine seems to play a central role in pair bond formation, expression, and maintenance [33]. *Zeb2* gene (*p* = 1.8 × 10^×153^), the top monogamy-related gene in our analysis, is also a key regulator of midbrain dopaminergic neuron development [34].

## Discussion

We expect that the availability of the human-*Peromyscus* epigenetic clocks will provide a significant boost to the attractiveness of *Peromyscus* as a biological model. This study provides a first step towards studying the epigenetic correlates of monogamous behavior and the effects of high altitude in *Peromyscus*.

The development of deer mouse epigenetic clocks described here was based on novel DNA methylation data that were derived from 3 deer mouse tissue types (brain, tail, and liver). The *Peromyscus* DNA methylation profiles reported here represent the most comprehensive dataset thus far of single-base resolution methylomes across multiple species, tissues, and ages.

The multi-tissue *Peromyscus* clock allows one to accurately estimate multiple tissue types from different *Peromyscus* species. This gives us confidence that these clocks will lead to high age correlations in new samples from other tissue types and from other *Peromyscus* species. However, differences in tissue type or species could lead to a constant offset (bias) between estimated age and actual age.

The two human-*Peromyscus* clocks estimate chronological and relative age, respectively. The dual-species clock for relative age demonstrates the feasibility of building epigenetic clocks for two species based on a single mathematical formula. These human-*Peromyscus* clocks also effectively demonstrate that epigenetic aging mechanisms are highly conserved between evolutionarily distant species. The fact that one can build such multispecies clocks reflects the technical properties of the mammalian methylation array platform (which focuses on conserved CpGs) [35] and biological properties of epigenetic aging effects that are highly conserved across mammals. Similar multispecies clocks have been constructed for many different mammalian species [36–41].

Our hierarchical clustering results reveal a robustly maintained hierarchical association of the biological variables (tissue, species) that can influence DNA methylation patterns. Tissue is a more important determinant of global methylation patterns than *Peromyscus* species (Fig. 1).

Our EWAS analyses identified CpGs associated with altitude at which the original founders of these colonies were captivated. These findings should be interpreted with caution because SM2 and BW stocks are highly diverse and are bred in isolation for extended periods which may have caused the fixation of methylation profiles that are irrelevant to the altitude adaptation of their original ancestors. Future studies should aim to replicate these results with wild-caught animals. While, however, no apparent differences have been recorded between the SM2 and the BW stocks that could associate with rhombomere 3 and motor neurons development, genes related to immune response and middle ear development may be of relevance. The latter may reflect adaptations associated with the differential atmospheric pressures at high altitudes. As regards the differential methylation of genes associated with immune system function, this may be relevant to the reported compromise of the immune system at high elevations [42–44]. To that end, high-altitude deer mice may have engaged epigenetic strategies to counteract such immune system suppression which has been linked, among others, to hypoxia at high elevations^27^. Of note is also the unique methylation profile of *P. maniculatus* in comparison with *P. leucopus* that corroborate the recently reported deregulation of stress response genes and the aberrant histological manifestations recorded in aged *P. maniculatus* [26].

Our EWAS of monogamy revealed strong methylation differences between monogamous and polygamous species. The fact that these results derived from tail tissues suggest that inherent differences in bonding behavior instruct specific epigenetic changes in peripheral tissues that may be translated into distinct physiological outcomes. Whether this is due to the differential regulation of specific neurohormonal circuits in response to hormones and neurotransmitters related to bonding, and which the exact physiological outputs are, remains to be determined.

Collectively, our study provided the first epigenetic clock for *Peromyscus* and illustrated the hierarchical association between various biological variables in determining methylation profiles across different scales of biological organization. Finally, it provided hints with regards to global differences and specific gene targets that are epigenetically impacted by biologically and environmentally relevant conditions.

## Materials and methods

Deer mice are maintained as outbred, genetically diverse closed colonies in the Peromyscus Genetic Stock Center (PGSC) of the University of South Carolina. The specific stocks used were *Peromyscus leucopus* (LL stock, white-footed mice), *Peromyscus eremicus* (EP stock, cactus, or desert mice), *Peromyscus polionotus* (PO stock, oldfield mice) and *Peromyscus manisculatus* (North American deer mice). For *P. maniculatus*, in particular, animals from two stocks were used, *P. maniculatus sonoriensis* (SM2, high altitude) and *P. manisculatus bardii* (BW stock, low altitude). F1 hybrids between BW and PO stocks were also used. More details about the specific stocks are available in ref (9) and on the PGSC website (https://go.sc.edu/pgsc). The study was approved by the Institutional Animal Care and Use Committee (IACUC) of the UofSC (protocol #: 2356–101,506-042,720) and was in accordance with the guidelines set forth by the National Institutes of Health. DNA was isolated from live animals by tail snips, or upon sacrifice from livers and brains by using DNeasy DNA isolation kit (Qiagen) using the manufacturer’s extraction. Our analysis is limited in that our brain data for P. leucopus and P. maniculatus derived from closed populations. The animals are technically outbred and genetic diversity is maintained, but there still may be a selection or drift over the several decades in captivity for/toward phenotypes that would otherwise be disadvantageous in the wild. Strictly speaking, our conclusions are limited to these closed colonies, which have low effective population sizes.

### Human tissue samples

To build the human-*Peromyscus* clock, we analyzed previously generated methylation data from *n* = 1205 human tissue samples (adipose, blood, bone marrow, dermis, epidermis, heart, keratinocytes, fibroblasts, kidney, liver, lung, lymph node, muscle, pituitary, skin, spleen) from individuals whose ages ranged from 0 to 93. These human tissue samples came from multiple sources: tissue and organ samples from the National NeuroAIDS Tissue Consortium [45], blood samples from the Cape Town Adolescent Antiretroviral Cohort study [46], and blood, skin, and other primary cells provided by Kenneth Raj [47] and blood samples from the PEG study [48]. Ethics approval was provided for all studies (IRB#15–001,454, IRB#16–000,471, IRB#18–000,315, IRB#16–002,028).

### DNA methylation data

The DNA methylation data were generated using the mammalian methylation array (HorvathMammalMethylChip40) based on 37,492 CpG sites [35]. Not all of these CpGs apply to deer mice. In our analysis, we focused on 29,125 CpGs that are located near 5048 genes in the deer mouse genome (Peromyscus_maniculatus_bairdii.HU_Pman_2.1.100).

Genome coordinates for each CpG are provided on the GitHub page of the Mammalian Methylation Consortium, see the section on data availability. The manifest file of the mammalian methylation array can be found at Gene Expression Omnibus (GEO) at NCBI as platform GPL28271. The SeSAMe normalization method was used to define beta values for each probe [49].

We caution the reader that small differences in sequences across species could have an effect on methylation levels.

To find technical outliers, we applied unsupervised hierarchical clustering (average linkage), which used 1 minus the interarray Pearson correlation as a dissimilarity measure. This analysis implicated five putative outliers which were severely outlying (i.e., that did not correlate with other samples from the same group). To err on the side of caution, we removed these samples from our analysis.

### Penalized regression models

Details on the clocks (CpGs, genome coordinates) and R software code are provided in the Supplement. Penalized regression models were created with glmnet [50]. We investigated models produced by both “elastic net” regression (alpha = 0.5). The optimal penalty parameters in all cases were determined automatically by using tenfold internal cross-validation (cv.glmnet) on the training set. By definition, the alpha value for the elastic net regression was set to 0.5 (midpoint between Ridge and Lasso type regression) and was not optimized for model performance.

We performed a cross-validation scheme for arriving at unbiased (or at least less biased) estimates of the accuracy of the different DNAm-based age estimators. One type consisted of leaving out a single sample (LOOCV) from the regression, predicting an age for that sample, and iterating over all samples. A critical step is the transformation of chronological age (the dependent variable).

Details on the clocks (CpGs, genome coordinates), coefficient values, and age transformations are provided in the Supplement.

To introduce biological meaning into age estimates of deer mice and humans that have a very different lifespan, as well as to overcome the inevitable skewing due to unequal distribution of data points from deer mice and humans across the age range, relative age estimation was made using the formula: relative age = age/maxlifespan where the maximum lifespan for the two species was chosen from the an age data base [23]. We used the following maximum lifespans *Peromyscus californicus* (5.5 years), *Peromyscus eremicus* (7.4 years), *Peromyscus leucopus* (7.9 years), *Peromyscus maniculatus* (8.3 years), *Peromyscus polionotus* (5.5 years), and humans (122.5 years), respectively.

### Epigenome-wide association studies of age

EWAS was performed in each tissue separately using the R function "standardScreeningNumericTrait" from the "WGCNA" R package [51]. We used Stouffer’s meta-analysis method to combine aging effects across different tissue types. Stouffer’s method forms a linear combination of the *Z*-scores which are calculated in each stratum (e.g., based on tissue type) [52]. We chose the same weight for each tissue type.

### GREAT analysis

We analyzed gene set enrichments using GREAT [53]. The GREAT enrichment analysis automatically conditioned out (removed) any bias resulting from the design of the mammalian array by using a background set of CpGs that map to horses and are located on the mammalian array. The GREAT software performs both a binomial test (over genomic regions) and a hypergeometric test over genes.

We performed the enrichment based on default settings (proximal: 50.0 kb upstream, 1.0 kb downstream, plus distal: up to 1000 kb) for gene sets implemented in GREAT. To avoid large numbers of multiple comparisons, we restricted the analysis to the gene sets with between 10 and 3000 genes. We report nominal *p*-values and two adjustments for multiple comparisons: Bonferroni correction and the Benjamini–Hochberg false discovery rate.

## Supplementary Information

Below is the link to the electronic supplementary material.

Supplementary file1 (DOCX 4027 KB)

## Data Availability

The data will be made publicly available as part of the data release from the Mammalian Methylation Consortium. Genome annotations of these CpGs can be found on Github https://github.com/shorvath/MammalianMethylationConsortium.
