# Supplementary material for: Methylation studies in Peromyscus: aging, altitude adaptation, and monogamy
Source: GeroScience. 2021 Oct 26;44(1):447–61. doi: 10.1007/s11357-021-00472-5 (PMC8810952; doi:10.1007/s11357-021-00472-5)
Supplement: Supplementary file 1 — Supplementary file1 (DOCX 4027 KB) [file 11357_2021_472_MOESM1_ESM.docx]

**SUPPLEMENTARY MATERIAL**


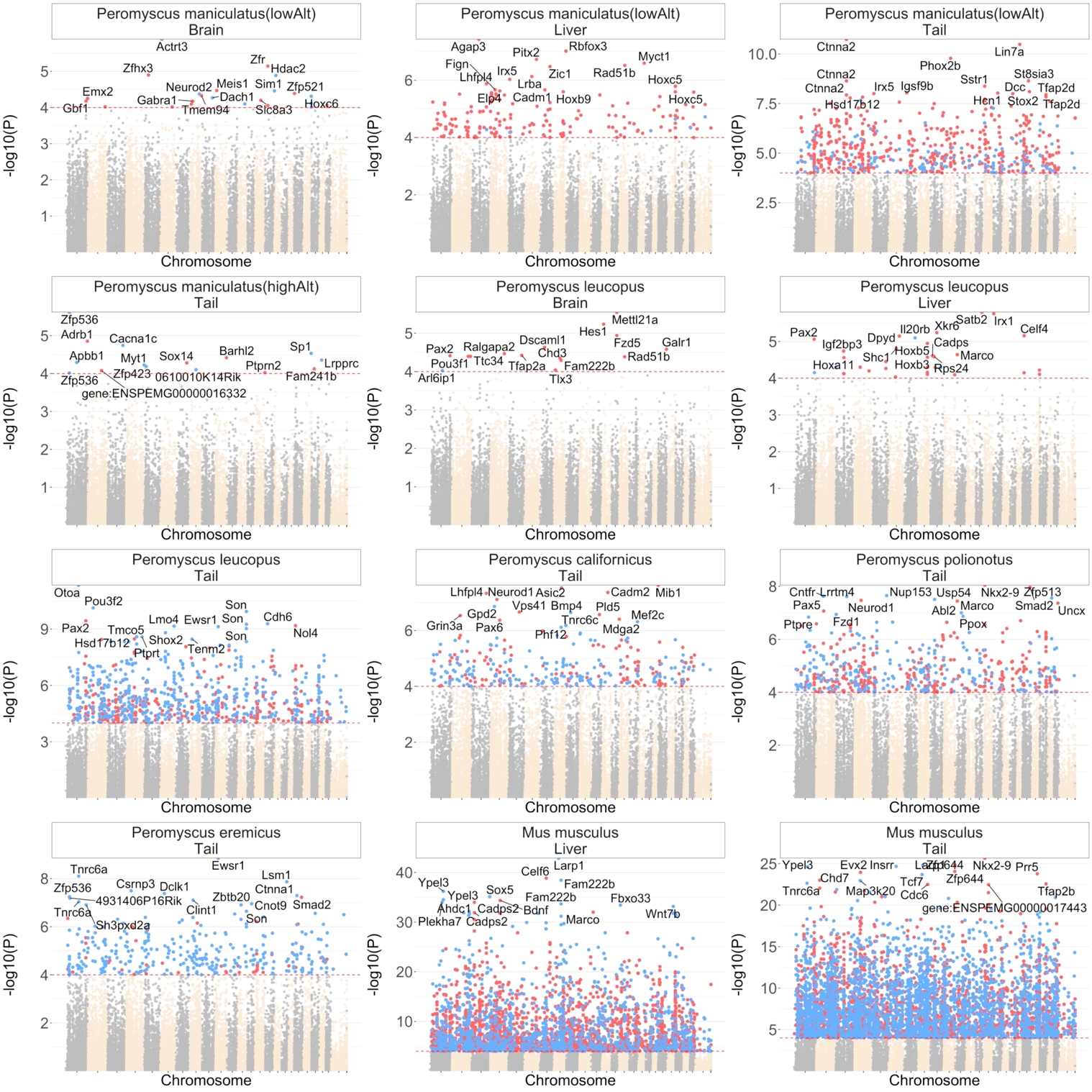


**Supplementary Figure 1.** **Manhattan plots of the** **EWAS of age in different tissues of *Peromyscus* genus and C57Bl/6 mouse.** The genome coordinates (x-axis) of the CpGs were derived from the genome assembly Peromyscus_maniculatus_bairdii.HU_Pman_2.1.100. The direction of associations with p < 10^-4^ (red dotted line) is colored in red (age related gain of methylation) and blue (age related loss of methylation). The top 15 most significant CpGs are labeled by the neighboring genes. The correlation test p-value (y-axis) has been transformed by minus logarithm (base 10).


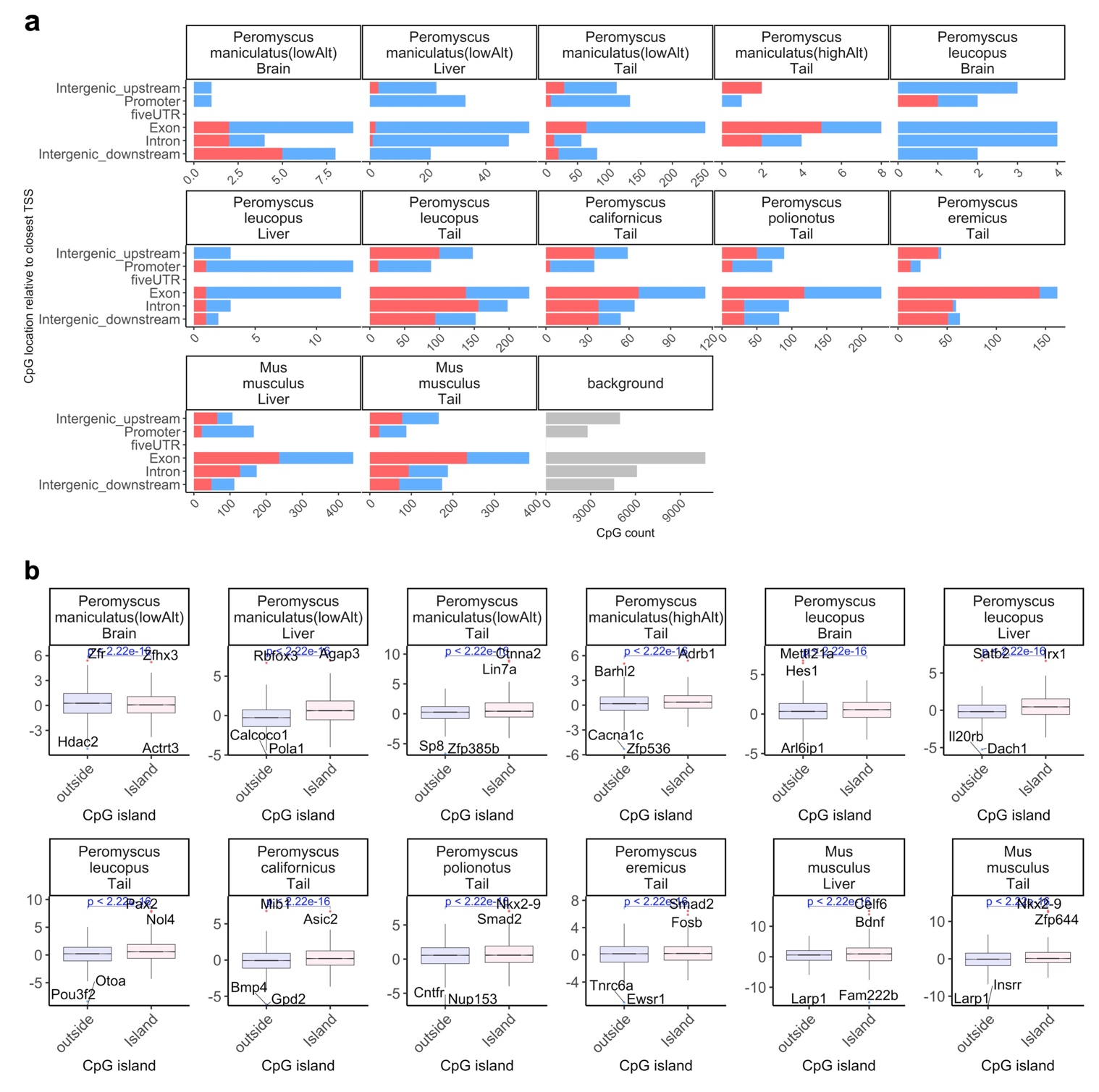


**Supplementary Figure 2.** **Gene region analysis of age effects on DNA methylation in *Peromyscus*.** a) Location of up to top 1000 (500 in each direction) significant CpGs in each species-tissue EWAS relative to the adjacent transcriptional start site. The grey color in the last panel represents the location of 29125 mammalian methylation array probes mapped to the Peromyscus_maniculatus_bairdii genome. b) Box plot analysis of aging effects on DNA methylation levels (Z statistics) by CpG island status. The top CpGs are labeled by adjacent genes. **** p<10^-4^. The x axis is the Fisher z-transformation of DNAm-Age Pearson correlation for each CpG in each tissue-species strata.


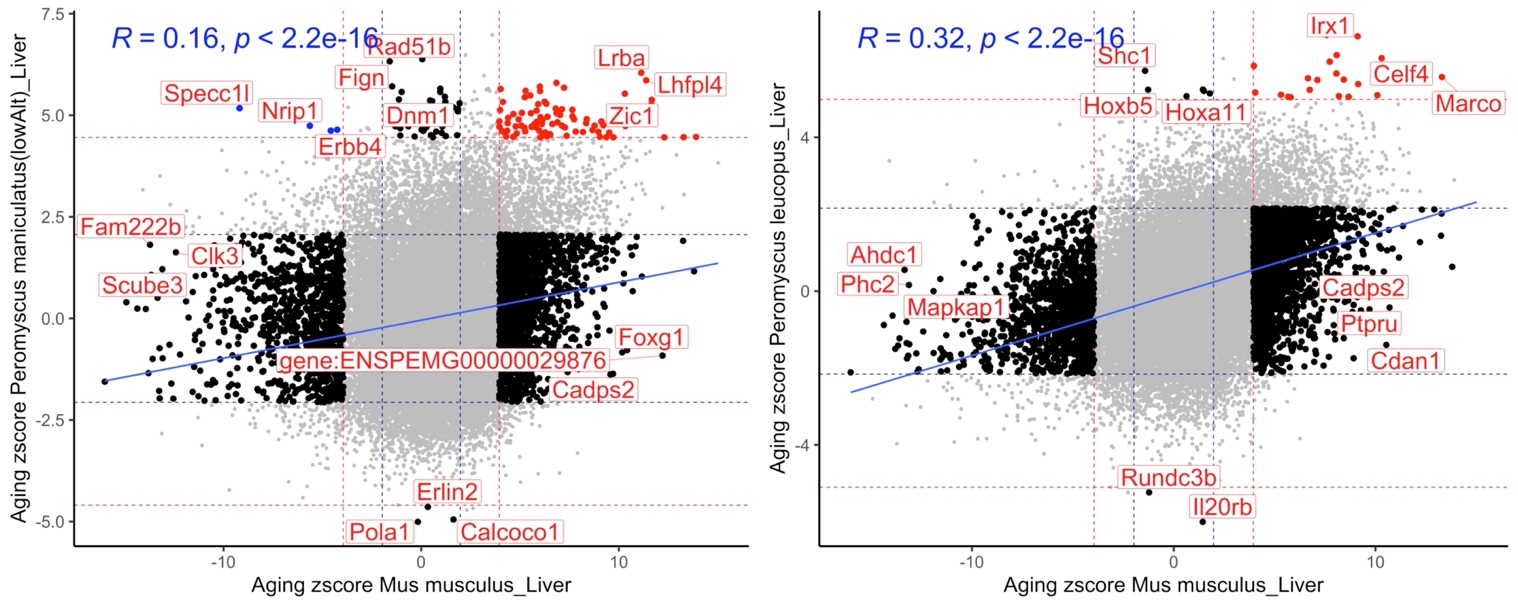


**Supplementary Figure 3. Liver** **DNAm aging in Peromyscus genus and C57Bl/6 mouse moderately correlate.** Sector plot of DNAm aging in Peromyscus species and C57Bl/6 mouse liver. Red dotted line: p<10^-4^; blue dotted line: p>0.05; Red dots: shared CpGs; black dots: species specific changes. The aging Z scores are the Fisher z-transformation of DNAm-Age Pearson correlation for each CpG in the liver samples of each species. Red dotted line are the Z scores corresponding to p<10^-4^; blue dotted line are the Z scores corresponding to p>0.05; Red dots indicate the shared CpGs (i.e. the CpGs that significantly change in the same direction) between X and Y axis; black dots: the changes that are significant in one species but not the other.


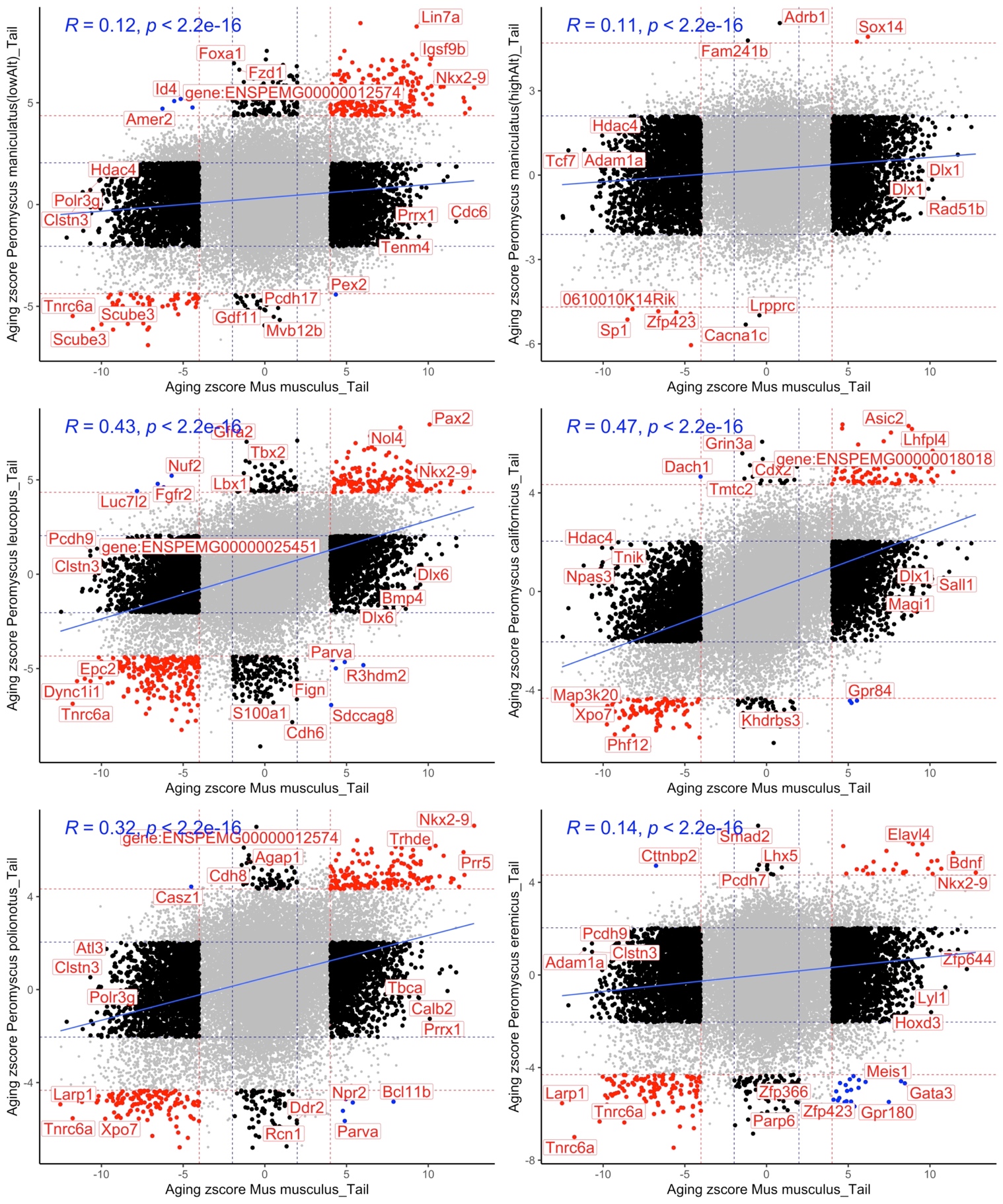


**Supplementary Figure 4. Comparison of DNAm aging in Peromyscus genus and C57Bl/6 mouse tail samples.** Sector plot of DNAm aging in Peromyscus species and C57Bl/6 mouse tails. The aging Z scores are the Fisher z-transformation of DNAm-Age Pearson correlation for each CpG in the tail samples of each species. Red dotted line are the Z scores corresponding to p<10^-4^; blue dotted line are the Z scores corresponding to p>0.05; Red dots indicate the shared CpGs (i.e. the CpGs that significantly change in the same direction) between X and Y axis; black dots: the changes that are significant in one species but not the other.


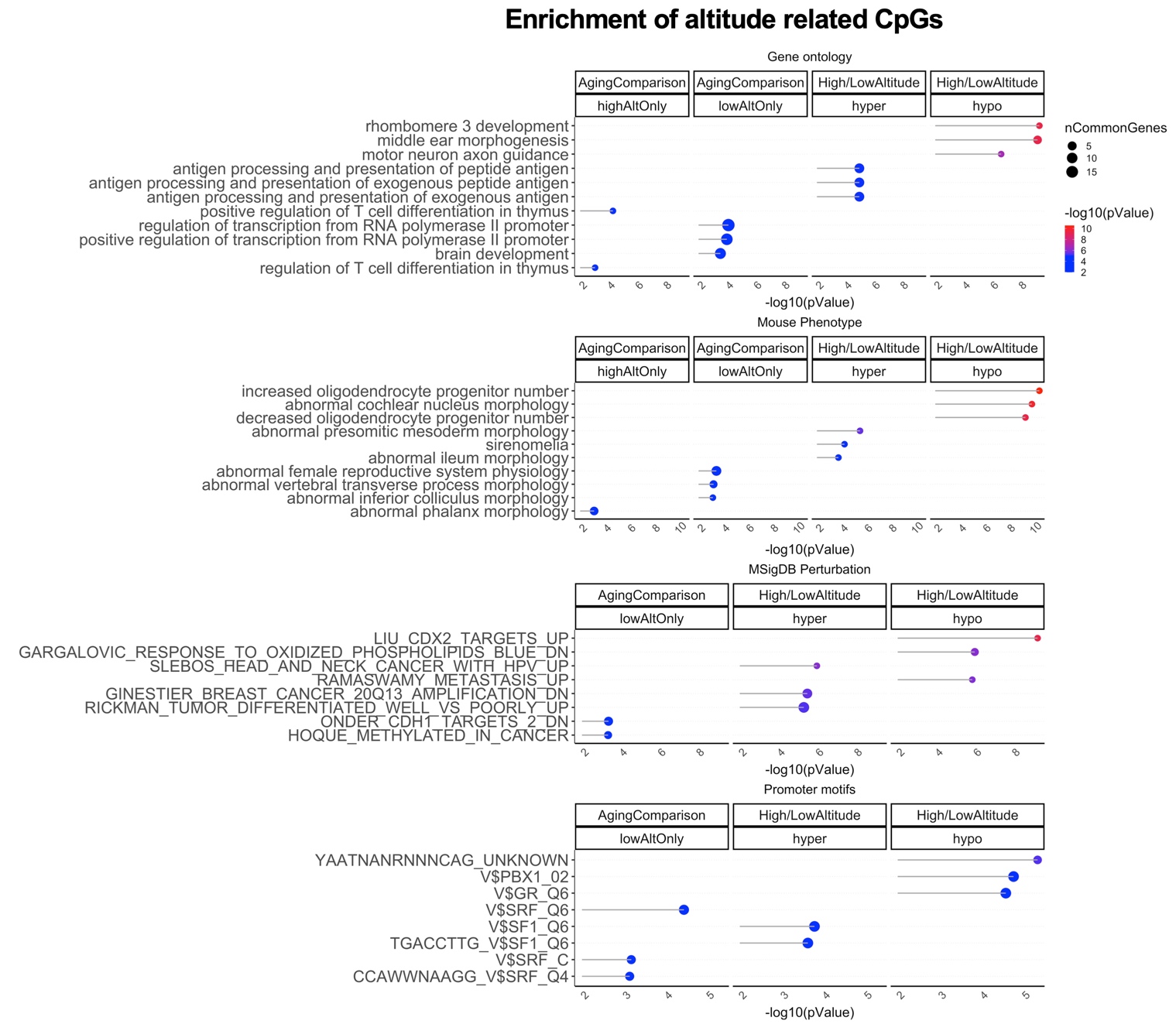


**Supplementary Figure 5.** **Gene set enrichment analysis of DNA methylation differences by altitude**. The gene level enrichment was done using GREAT analysis [1] and human Hg19 background. The CpGs were annotate with adjacent genes in 50kb flanking region. We extracted up to top 500 CpGs based on pvalue of association per direction of change as input for the enrichment analysis (e.g. CpGs that have a higher or lower DNAm levels in high altitude *P. maniculatus* vs the low altitude *P. maniculatus*). The pvalues are calculated by hypergeometric test of the EWAS results with the genes in each background dataset. The background was limited to the genes adjacent to 20,515 CpGs that are conserved between *Peromyscus maniculatus* and human annotations. Datasets: gene ontology, mouse phenotypes, promoter motifs, and MsigDB Perturbation, which includes the expression signatures of genetic perturbations curated in GSEA database [2]. The results were filtered for significance at p < 10^-3^ and only the top terms for each EWAS result.


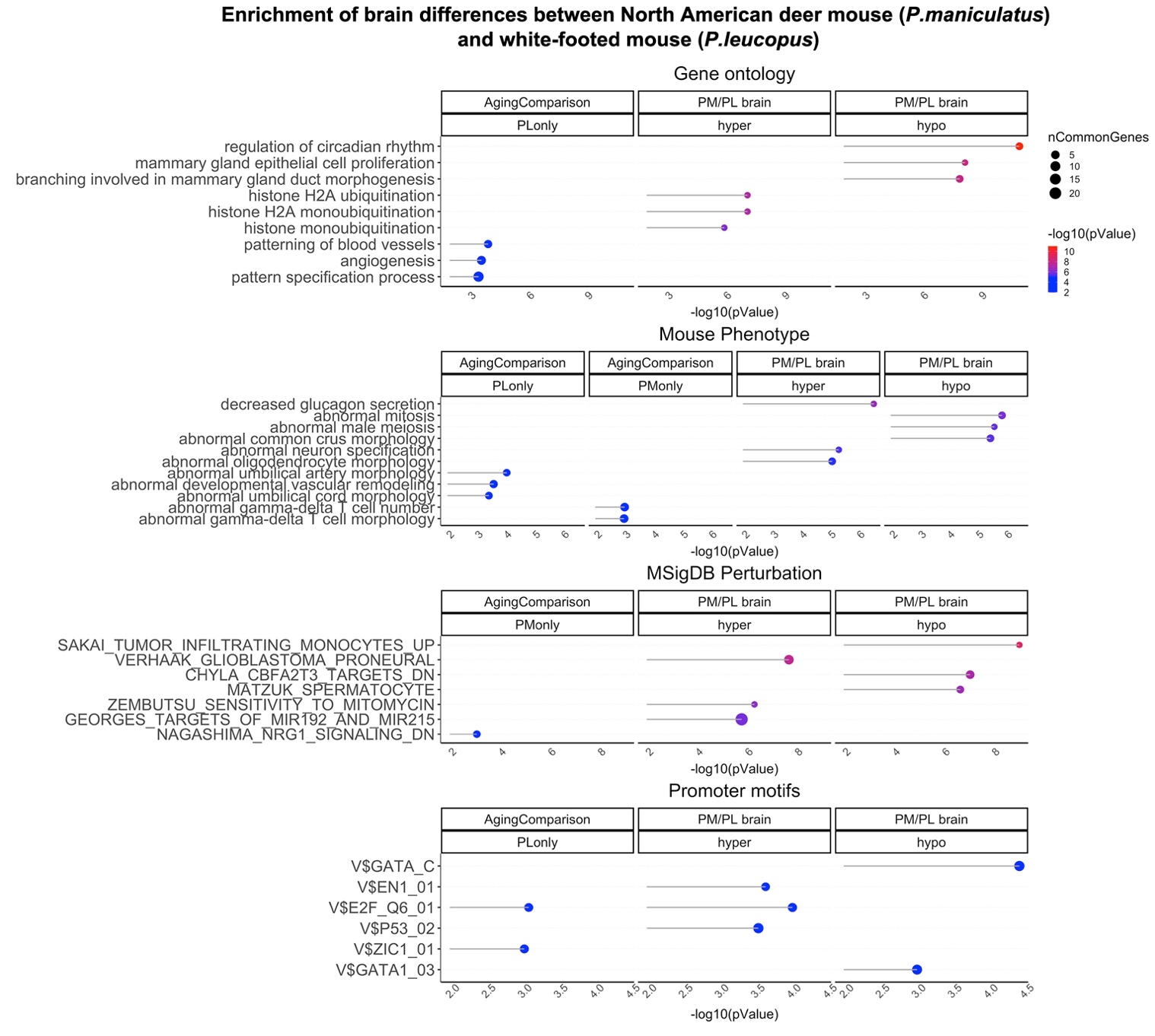


**Supplementary Figure 6. Gene set enrichment analysis of DNA methylation differences between *P. maniculatus* and *P. leucopus* brains.** The gene level enrichment was carried out with GREAT analysis [1] and human Hg19 background. The CpGs were annotate with adjacent genes in 50kb flanking region. We extracted up to top 500 CpGs based on pvalue of association per direction of change as input for the enrichment analysis (e.g. CpGs that have a higher or lower DNAm levels in P. maniculatus vs P. leucopus). The pvalues are calculated by hypergeometric test of the EWAS results with the genes in each background dataset. The background was limited to the genes adjacent to 20,515 CpGs that are conserved between *Peromyscus maniculatus* and human annotations. Datasets: gene ontology, mouse phenotypes, promoter motifs, and MsigDB Perturbation, which includes the expression signatures of genetic perturbations curated in GSEA database [2]. The results were filtered for significance at p < 10^-3^ and only the top terms for each EWAS result. PM, P. maniculatus; PL, P. leucopus. The column "AgingComparison" report results for CpGs that change with age only in one species but not the other.


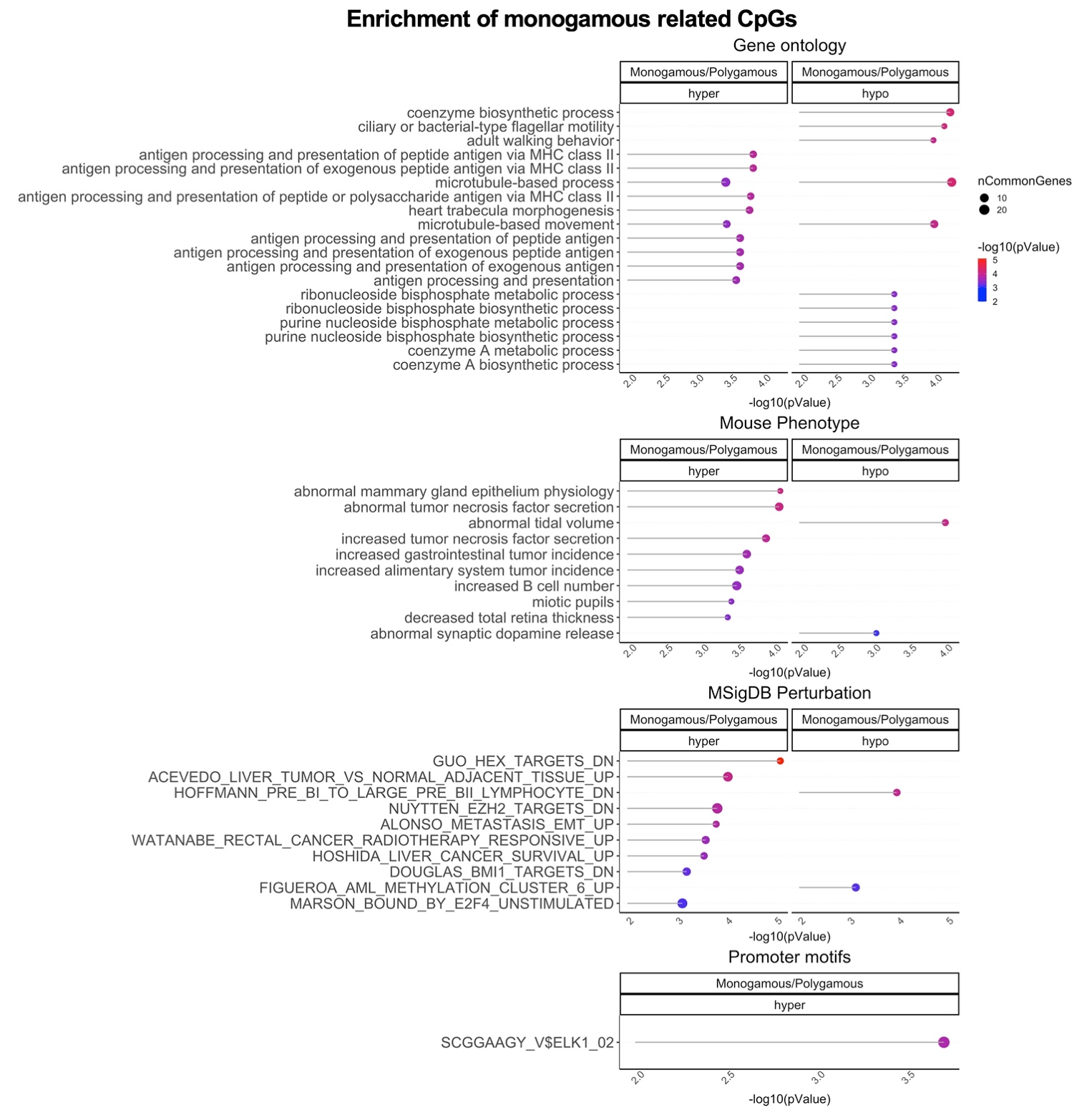


**Supplementary Figure 7.** **Gene set enrichment analysis of DNA methylation differences between monogamous and polygamous *Peromyscus* species.** The gene level enrichment was done using GREAT analysis [1] and human Hg19 background. The CpGs were annotate with adjacent genes in 50kb flanking region. We extracted up to top 500 CpGs based on pvalue of association per direction of change as input for the enrichment analysis (e.g. CpGs that have a higher or lower DNAm levels in monogamous vs polygamous species). The pvalues are calculated by hypergeometric test of the EWAS results with the genes in each background dataset. The background was limited to the genes adjacent to 20,515 CpGs that are conserved between *Peromyscus maniculatus* and human annotations. Datasets: gene ontology, mouse phenotypes, promoter motifs, and MsigDB Perturbation, which includes the expression signatures of genetic perturbations curated in GSEA database [2]. The results were filtered for significance at p < 10^-3^ and only the top terms for each EWAS result.

**Technical Details surrounding the DNAm age estimator**

**Statistical methods used for building the clocks**

The epigenetic clocks were used by employing a single elastic net regression model analysis (R function glmnet). We use used Leave-one-out analysis (LOO) using a single lambda value. We chose the following parameters for the glmnet R function (Alpha: 0.5, CV Fold: 10, Lambda choice for Clock: 1 standard error above minimum CV-MSE).

**Covariates and coefficient values of the peromyscusclocks**

1. The peromyscuspan tissue clock is based on 45 CpGs whose coefficient values are specified in the column "Coef.PeromyscusMultiTissue". Age transformation=identity, i.e. F(Age)=Age
2. The peromyscus Tail tissue clock is based on 34 CpGs whose coefficient values are specified in the column "Coef.PeromyscusTail". Age transformation=identity, i.e. F(Age)=Age
3. The peromyscus Liver tissue clock is based on 35 CpGs whose coefficient values are specified in the column "Coef.PeromyscusTail". Age transformation=identity, i.e. F(Age)=Age
4. The peromyscus Brain tissue clock is based on 15 CpGs whose coefficient values are specified in the column "Coef.PeromyscusTail". Age transformation=identity, i.e. F(Age)=Age
5. The Human Peromyscus clock for chronological age is based on 530 CpGs whose coefficient values are specified in the column "Coef.HumanPeromyscusLogLinearAge". Age transformation=log-linear described below.
6. The final human peromyscusclock for relative age is based on 578 CpGs whose coefficient values are specified in the column "Coef.HumanPeromyscusRelativeAge". Age transformation: relative age. i.e. F(Age)=Age/maxLifespan. Max lifespan for peromyscus can be found in the main text. Human max lifespan =122.5 years. According to the data base "anAge" [3], the maximum lifespans are as follows: 7.4 years for Peromyscus eremicus [4], 7.9 years for Peromyscus leucopus [4], 8.3 years for Peromyscus maniculatus [5], and 5.5 years for Peromyscus polionotus [4]. While the maximum lifespan estimates may be debatable, our clock is quite robust with respect to different choices of this mathematical parameter. Similarly accurate clocks could be constructed with different parameter choices.

**General description of age transformation**

The human-*Peromyscus* clocks for chronological age used log linear transformations that are similar to those employed for the HUMAN pan tissue (Horvath 2013) [6].

An elastic net regression model (implemented in the glmnet R function) was used to regress a transformed version of age on the beta values in the training data. The glmnet function requires the user to specify two parameters (alpha and beta). Since I used an elastic net predictor, alpha was set to 0.5. But the lambda value of was chosen by applying a 10 fold cross validation to the training data (via the R function cv.glmnet).

The elastic net regression results in a linear regression model whose coefficients b_0_, b_1_, . . . , relate to transformed age as follows
*F*(chronological age)=*b*_0_*+b*_1_*CpG*_1_*+ . . . +b*_p_*CpG*_p_+error

Note that the intercept term is denoted by b_0_. The coefficient values can be found in the attached Excel file.

Based, on the coefficient values from the regression model, DNAmAge is estimated as follows
*DNAm*Age=$F^{-1}$(*b*_0_*+b*_1_*CpG*_1_*+ . . . +b*_p_*CpG*_p_)

where $F^{-1}\left( y \right)$ denotes the mathematical inverse of the function F(.). Thus, the regression model can be used to predict to transformed age value by simply plugging the beta values of the selected CpGs into the formula.

In order to use this transformation to predict Age on *new samples*, one needs to use the *inverse* transformation, F^-1^(y), given by

$$F^{-1}\left( y \right)= \left\{ \begin{aligned} \begin{aligned} \begin{aligned} \left( A+1.5 \right)*\text{exp}\left( y \right)-1.5, for y\leq0 \\ (A+1.5)y+A, for y\geq0 \end{aligned} \end{aligned} \end{aligned} \right.$$

For predicting age, apply the inverse transformation to coefficient-weighted sum. That is,

$$DNAmAge=F^{-1}\left( x*\beta\right)$$

where $\beta$ is the vector of coefficients and $x$ is the vector of methylation values, with an intercept term.

For the human-peromyscus clocks we used the following averages at sexual maturity (in units of years): 13.5 years for humans and 0.353424658 years for peromyscus

## **The DNAm Age estimate is estimated in two steps.**

First, one forms a weighted linear combination of the CpGs whose details can be found in Table

The table reports the probe identifier (cg number) used in the custom Infinium array (HorvathMammalMethylChip40) . The weights used in this linear combination are specified in the respective column entitled "Coef.".

The formula assumes that the DNA methylation data measure "beta" values but the formula could be adapted to other ways of generating DNA methylation data.

**References:**

[1] C. Y. McLean, D. Bristor, M. Hiller, S. L. Clarke, B. T. Schaar, C. B. Lowe*, et al.*, "GREAT improves functional interpretation of cis-regulatory regions," *Nat Biotechnol,* vol. 28, pp. 495-501, May 2010.

[2] A. Subramanian, P. Tamayo, V. Mootha, S. Mukherjee, B. Ebert, M. Gillette*, et al.*, "Gene set enrichment analysis: A knowledge-based approach for interpreting genome-wide expression profiles," *Proceedings of the National Academy of Sciences of the United States of America,* vol. 102, pp. 15545-15550, 2005.

[3] J. P. de Magalhaes, J. Costa, and G. M. Church, "An analysis of the relationship between metabolism, developmental schedules, and longevity using phylogenetic independent contrasts," *J Gerontol A Biol Sci Med Sci,* vol. 62, pp. 149-60, Feb 2007.

[4] R. Weigl, "Longevity of mammals in captivity; from the living collections of the world," 2005.

[5] R. M. Nowak and E. P. Walker, *Walker's Mammals of the World* vol. 1: JHU press, 1999.

[6] S. Horvath, "DNA methylation age of human tissues and cell types," *Genome Biol,* vol. 14, p. R115, 2013.
